# Preventive effects of active Protocol-Based Pharmacotherapy Management for delirium in general wards of emergency department (aPBPM trial): study protocol for a single-center, randomized controlled trial {1a}

**DOI:** 10.1186/s13063-026-09781-6

**Published:** 2026-05-20

**Authors:** Yudai Takatani, Yuki Sato, Naoko Sugita, Hiroyasu Abe, Mika Yamane, Yuichi Yamanaka, Masumi Takeda, Takuma Minami, Tomohiro Terada, Shigeru Ohtsuru

**Affiliations:** 1 Department of Primary Care and Emergency Medicine, Graduate School of Medicine, Kyoto University, Kyoto, Japan; 2 Department of Clinical Pharmacology and Therapeutics, Kyoto University Hospital, Kyoto, Japan; 3 Department of Psychiatry, Kyoto University Hospital, Kyoto, Japan; 4 Department of Psychiatry, National Hospital Organization Kyoto Medical Center, Kyoto, Japan; 5 School of Pharmaceutical Sciences, Wakayama Medical University, Wakayama, Japan; 6 Department of Nursing, Kyoto University Hospital, Kyoto, Japan

**Keywords:** Delirium, Prevention, Protocol-Based Pharmacotherapy Management (PBPM), Emergency medicine, Elderly, Pharmacist intervention, Randomized controlled trial

## Abstract

**Background:**

Delirium is a frequent complication in older patients admitted to emergency wards, leading to prolonged hospitalization and increased mortality. Although multicomponent interventions are recommended, the evidence for pharmacological prevention remains inconsistent. Protocol-Based Pharmacotherapy Management (PBPM) allows pharmacists to actively manage medications according to a pre-agreed protocol. This study aims to evaluate the efficacy and safety of an “Active PBPM” strategy for delirium prevention compared with that via a standard “Passive PBPM” strategy in elderly emergency patients.

**Methods:**

This single-center, open-label, randomized controlled trial will target patients aged ≥65 years admitted to the general ward of the Department of Primary Care and Emergency Medicine from the emergency room. Eligible patients must have at least one risk factor (e.g., cognitive dysfunction, central nervous system disease). The patients will be randomized (1:1) into either the Active or Passive PBPM group. In the Active PBPM group, pharmacists will intervene to administer prophylactic medication for 3 days, starting on admission day 1. Patients capable of oral intake will receive ramelteon (8 mg) and lemborexant (5 mg) at bedtime. Patients unable to take oral medication will receive a blonanserin patch (20 mg) applied overnight (19:00–06:00). In the Passive PBPM group, pharmacological intervention will be performed only if insomnia occurs. The primary outcome will be the incidence of delirium diagnosed by a psychiatrist based on the Diagnostic and Statistical Manual of Mental Disorders, 5th Edition criteria, within the first 3 days. The secondary outcomes include delirium severity (Delirium Rating Scale-Revised-98), assessed by trained nurses, and safety.

**Discussion:**

This study will determine whether pharmacist-led proactive pharmacological intervention (Active PBPM) reduces the incidence of delirium in high-risk older patients in acute care settings. The findings may establish a new standard of care for the prevention of delirium in emergency medicine.

**Trial registration:**

Japan Registry of Clinical Trials (jRCT) jRCTs051220122. Registered on November 25, 2022

## Protocol version {2}

The current protocol version is 6.0, dated May 26, 2025.

## Background

### Background and rationale {9a}

Delirium is an acute neuropsychiatric syndrome associated with significant adverse outcomes, including prolonged hospitalization, increased mortality, and functional decline [1]. Older patients admitted to emergency departments are at particularly high risk of delirium because of the severity of acute intrinsic and extrinsic diseases, making the prevention of delirium a critical priority in acute care medicine [2].

Current guidelines recommend multicomponent non-pharmacological interventions (e.g., reorientation, early mobilization) as first-line strategies [3, 4]. However, the consistent implementation of these labor-intensive strategies is often challenging in busy clinical settings. Therefore, pharmacological prophylaxis has become an area of interest. In intensive care units (ICUs) and high-care units (HCUs), the alpha-2 agonist dexmedetomidine has garnered attention for delirium prevention [5]. We previously investigated the utility of dexmedetomidine in an HCU setting [6]. However, the use of continuous intravenous sedatives, such as dexmedetomidine, is generally not indicated or feasible in general wards because of the requirement for strict hemodynamic monitoring.

This creates a therapeutic gap for patients in general wards. Unlike ICUs, general wards typically have lower nurse-to-patient ratios, making early detection and management of delirium paradoxically more challenging. Therefore, a safe and effective pharmacological strategy suitable for general ward settings is urgently required. In our clinical practice, we use melatonin receptor agonists (ramelteon), which have some evidence for delirium prevention [7, 8], in combination with dual orexin receptor antagonists (lemborexant), which are known for their favorable safety profile and low risk of inducing delirium [9]. Furthermore, for patients unable to take oral medications, we use a blonanserin patch, a transdermal antipsychotic. Blonanserin is primarily available in Japan and other Asian countries, and its transdermal formulation has been developed specifically in Japan [10]. Although the blonanserin patch is officially indicated for schizophrenia, it is widely used off-label for the treatment of delirium in clinical practice, including at our institution, because of its favorable safety profile and ease of use in patients with swallowing difficulties [11]. However, its efficacy in prevention has not yet been established. This study aims to reposition this agent for prophylactic use.

### Explanation for the choice of comparator {9b}

To consistently implement this multimodal strategy, we focus on the role of pharmacists. In the United States, Collaborative Drug Therapy Management allows pharmacists to actively manage medication therapy [12]. In Japan, a similar system, the Protocol-Based Pharmacotherapy Management (PBPM), has been gaining recognition. We have recently reported the efficacy of PBPM in optimizing antimicrobial therapy [13].

### Objectives {10}

We aim to evaluate the efficacy and safety of a pharmacist-led “Active PBPM” strategy for delirium prevention compared with standard care in elderly emergency patients admitted to general wards.

## Methods: patient and public involvement, and trial design

### Patient and public involvement {11}

No patients or members of the public were involved in the design, conduct, reporting, or dissemination plans of this study. The research protocol was developed by a multidisciplinary team of medical professionals (emergency physicians, psychiatrists, and pharmacists) based on clinical necessity and existing guidelines to ensure patient safety and feasibility in emergency settings.

### Trial design {12}

This single-center, prospective, randomized, open-label, parallel-group, interventional study is designed as a superiority trial. It aims to test the hypothesis that participants in the active PBPM group will have a lower incidence of delirium than those in the passive PBPM group.

A multidisciplinary team will collaborate with clearly defined roles to ensure comprehensive delirium prevention and management:

- Nurses: Responsible for daily delirium screening using the Delirium Rating Scale-Revised-98 (DRS-R98-J) [14], monitoring sleep–wake cycles, and identifying potential adverse events.
- Pharmacists: Review medication history and brought-in drugs upon admission to identify high-risk medications, and implement the PBPM for delirium risk reduction.
- Emergency physicians: Act as attending physicians, supervise overall clinical management, and facilitate the implementation of PBPM interventions in collaboration with pharmacists.
- Psychiatrists: Provide definitive diagnosis of delirium according to the Diagnostic and Statistical Manual of Mental Disorders, 5th Edition (DSM-5) criteria [15] and provide expert consultation for severe or refractory cases.

## Methods: participants, interventions and outcomes

### Trial setting {13}

This study will be conducted in the general ward of the Department of Primary Care and Emergency Medicine at Kyoto University Hospital (Kyoto, Japan).

### Characteristics of the people who are needed for the trial

**Table:** 

**Characteristic**	**The people we would expect to see included**
Age	≥ 65 years
Sex	No restriction
Gender	No restriction
Race, ethnicity and ancestry	No restriction
Socioeconomic status	No restriction
Geographic location	Kyoto, Japan
Other characteristics relevant to the trial	Present with at least one of the following six delirium risk factors, based on the Japanese national medical reimbursement criteria (“Delirium High-Risk Patient Management Addition”). Importantly, these factors are consistent with the established predisposing factors identified in major epidemiological studies [3, 16] and with international standards (e.g., American Geriatrics Society Beers Criteria) [17]: ・Central nervous system diseases (e.g., cerebrovascular disease, traumatic brain injury) ・Cognitive dysfunction ・History of high alcohol intake (defined as ≥ 4 days/week) ・History of delirium ・Current use of delirium-inducing drugs (e.g., benzodiazepines, corticosteroids, opioids, H2-receptor antagonists, psychotropics, anti-parkinsonian agents, or anticholinergics) ・Post-surgery or scheduled surgery requiring general anesthesia

### Eligibility criteria for participants {14a}

Patients will be eligible if they meet all the following inclusion criteria:

1. Admitted to the general ward of the Department of Primary Care and Emergency Medicine via the emergency department
2. Age ≥ 65 years
3. Present with at least one of the following six delirium risk factors, based on the Japanese national medical reimbursement criteria (“Delirium High-Risk Patient Management Addition”). Importantly, these factors are consistent with the established predisposing factors identified in major epidemiological studies [3, 16] and with international standards (e.g., American Geriatrics Society Beers Criteria) [17]:
4. Central nervous system diseases (e.g., cerebrovascular disease, traumatic brain injury)
5. Cognitive dysfunction
6. History of high alcohol intake (defined as ≥4 days/week)
7. History of delirium
8. Current use of delirium-inducing drugs (e.g., benzodiazepines, corticosteroids, opioids, H2-receptor antagonists, psychotropics, anti-parkinsonian agents, or anticholinergics)
9. Post-surgery or scheduled surgery requiring general anesthesia

Patients will be excluded if they meet any of the following criteria:

1. Schizophrenia
2. Taking antipsychotics
3. Unable to be hospitalized for >48 h
4. Taking benzodiazepines prescribed by a psychiatrist or psychosomatic physician
5. Taking CYP3A4 inhibitors (e.g., itraconazole, clarithromycin, erythromycin, fluconazole, verapamil, human immunodeficiency virus protease inhibitors)
6. Taking ramelteon or lemborexant
7. Blonanserin patch is already applied
8. Severe liver dysfunction
9. Severe disturbance of consciousness
10. Sedatives are used before the start of the study
11. Drug abuse

### Eligibility criteria for sites and those delivering interventions {14b}

This study will be conducted at a single tertiary care academic hospital (Kyoto University Hospital). The interventions are delivered by licensed medical professionals (physicians, pharmacists, and nurses) affiliated with the Department of Primary Care and Emergency Medicine. All participating pharmacists are trained in the specific PBPM protocol, and psychiatrists provide expert diagnostic confirmation.

### Who will take informed consent? {32a}

Written informed consent will be obtained from all participants. Given the emergency setting, if a participant lacks the capacity to provide consent owing to impaired consciousness or cognitive dysfunction, written informed consent will be obtained from a legally authorized representative (e.g., spouse, parent, or adult child). If the participant regains capacity, they will be informed about the study, and their consent for continued participation will be obtained. To ensure privacy, all personal information will be pseudonymized using participant ID codes.

### Additional consent provisions for collection and use of participant data and biological specimens {32b}

The link between the ID codes and identifiable personal information will be stored in a secure, locked location at the study site. Access to the raw data will be restricted to authorized study personnel only. All data will be maintained in accordance with the CRA and the study protocol.

## Intervention and comparator

### Intervention and comparator description {15a}

**Fig. 1:**
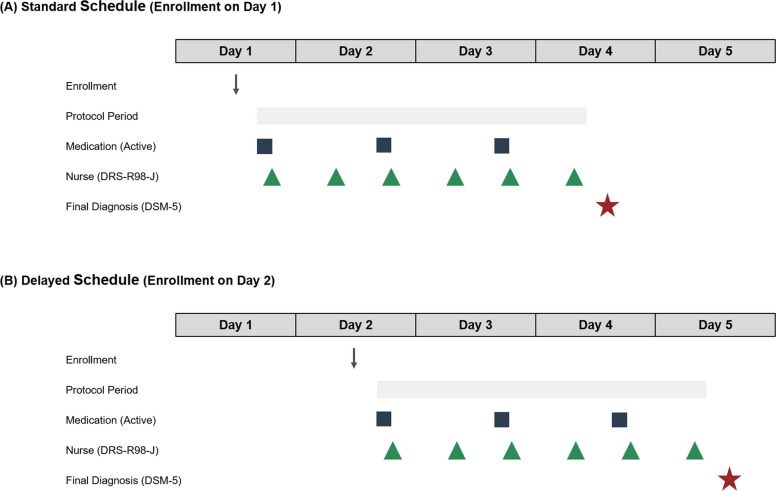
Detailed intervention and assessment schedules by enrollment timing. The timeline varies depending on the timing of enrollment. **A **Standard schedule: Enrollment on day 1 (08:30–17:00), with the 72-h protocol period starting that night. **B **Delayed schedule: Enrollment on day 2 for patients admitted at night or outside regular hours. The gray bar represents the 72-h protocol period. Blue squares indicate medication administration at 19:00 (Active group only). Green triangles indicate twice-daily nurse assessments using the DRS-R98-J at 20:00 and 10:00 (6 assessments total). The red star indicates the final diagnosis of the primary outcome by a blinded psychiatrist using DSM-5 criteria, performed within 3 days after protocol completion

The intervention period will be 3 days. The detailed protocols for the study medications are summarized in Table 1 and Fig. 1.

**Table 1:** Protocol for study medications in the Active and Passive PBPM groups

Group	Route	Scheduled medication(19:00)	Action on insomnia
Active PBPM	Oral	**Ramelteon** 8 mg + **lemborexant** 5 mg	Additional **lemborexant** 5 mg (if insomnia persists)
	Non-oral	**Blonanserin** **patch** 20 mg (19:00–06:00)	**Hydroxyzine** (rescue)
Passive PBPM	Oral	None	1. **Ramelteon** 8 mg + **lemborexant** 5 mg 2. Additional **lemborexant** 5 mg (if insomnia persists) (Switch to regular **ramelteon** from next day)
	Non-oral	None	**Hydroxyzine** (rescue)

Intervention period: The protocol-based intervention will be performed for 3 consecutive days from the date of enrollment (day 1 to day 3). Active PBPM group: Pharmacists will actively intervene to initiate prophylactic medications, regardless of the presence of symptoms. Passive PBPM group: Medications will be administered only as symptomatic treatment if insomnia or delirium symptoms are observed. Regular administration in the Passive group: If rescue medication is required for insomnia in the oral intake group, Ramelteon 8 mg will be switched to regular administration at 19:00 from the following day.・Blonanserin patch: In the Active PBPM (non-oral) group, the patch will be applied for 11 h (from 19:00 to 06:00 the following morning)

*Abbreviations*: *PBPM* Protocol-Based Pharmacotherapy Management; *eGFR* estimated glomerular filtration rate

1. Active PBPM group: Pharmacists will screen for delirium-inducing drugs upon admission. The patients will receive prophylactic medications for three consecutive nights.
  Oral intake possible: Ramelteon and lemborexant daily at 19:00.
  Oral intake impossible: The blonanserin patch will be applied daily from 19:00 to 06:00.
2. Passive PBPM group: No prophylactic medications will be administered. Medications will be provided as rescue therapy only if insomnia occurs, following standard care procedures (Table 1).

### Criteria for discontinuing or modifying allocated intervention/comparator {15b}

Interventions will be discontinued in case of (1) intolerable adverse events, (2) use of prohibited medications, (3) withdrawal of consent, (4) post-enrollment discovery of ineligibility, or (5) significant clinical deterioration or death. No interim analysis is planned for this study. As such, no formal stopping rules for the entire trial have been established based on efficacy or futility. However, the principal investigator may terminate the trial early if unforeseen safety concerns arise.

### Strategies to improve adherence to intervention/comparator {15c}

Adherence to the study protocol is rigorously maintained through the structured PBPM framework. In this system, pharmacists proactively identify eligible patients and propose medication plans based on the pre-agreed protocol. These plans are then reviewed and formally approved by the attending physicians before any treatment is implemented. This collaborative verification process ensures that the intervention strictly adheres to the trial design. Furthermore, all prescriptions and administrations are recorded in the electronic medical record system, which is regularly reviewed by the research team to monitor and ensure full compliance with the protocol.

### Concomitant care permitted or prohibited during the trial {15d}

#### Standard care and concomitant management

All participants will receive standard non-pharmacological delirium prevention, including cognitive orientation, dehydration prevention, early mobilization, and sleep hygiene.

**Fig. 2:**
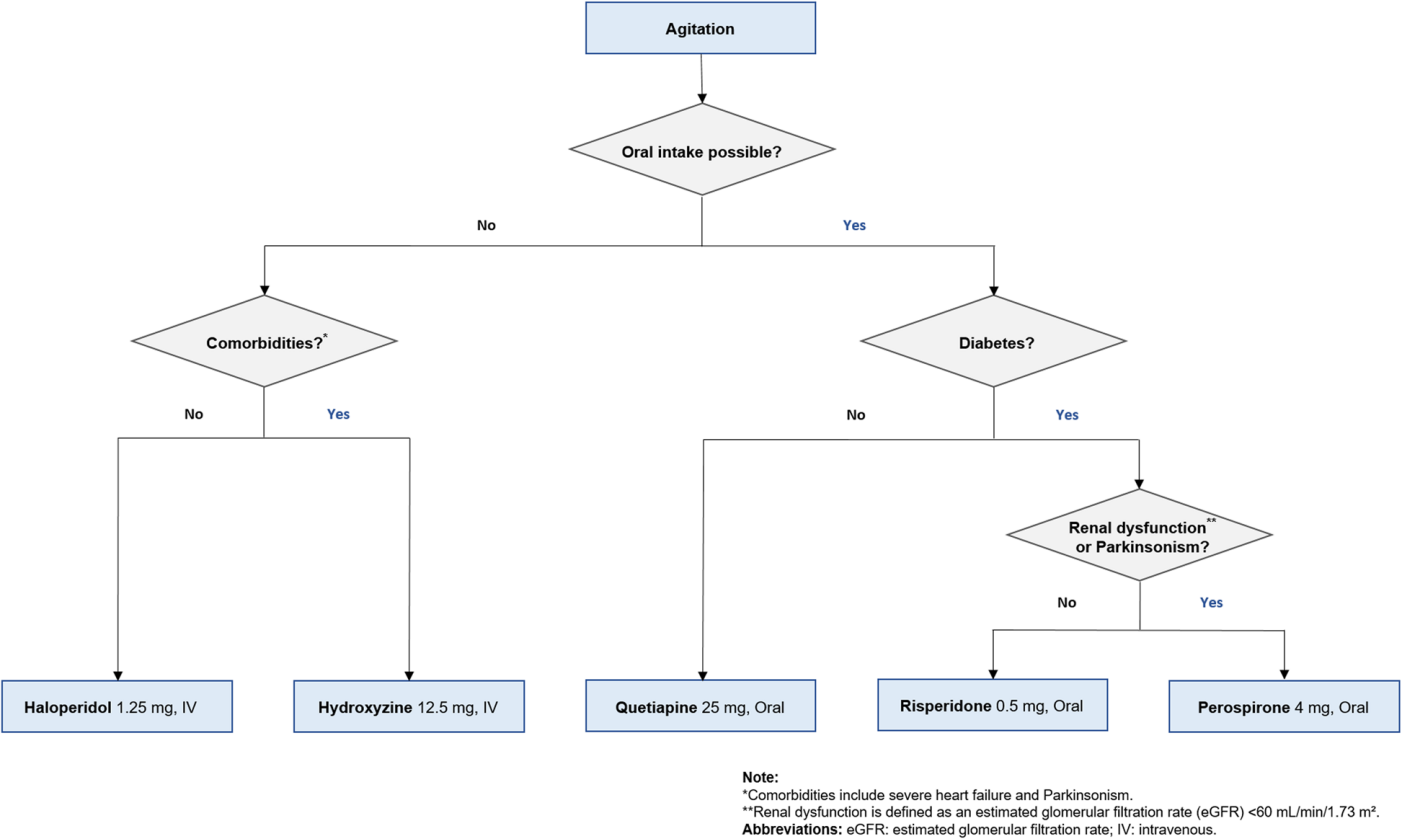
Standardized pharmacological management for agitation. Standardized pharmacological management flow when agitation develops during the study period

- Rescue medications: If agitation develops, rescue medications (e.g., intravenous hydroxyzine or haloperidol) will be administered according to a standardized treatment algorithm (Fig. 2).
- Prohibited medications: Concomitant use of other antipsychotics or hypnotics will be strictly prohibited during the study period.
- Adverse events: Any adverse events will be managed with appropriate supportive care. Detailed procedures for the systematic collection and classification of harms are described in the Harms {17} section. The participants will be covered by the clinical trial insurance.

### Ancillary and post-trial care {34}

As mentioned in the Concomitant care section {15d}, participants are covered by clinical trial insurance for any study-related harm. After the 3-day study period, patients in the Active PBPM group will transition to the Passive PBPM protocol (standard management), while those in the Passive PBPM group will continue their current management. All other medical treatments and follow-up care will be provided at the discretion of the attending or sub-investigators based on the patients’ clinical needs.

### Outcomes {16}

The primary and secondary outcomes are defined below. Table 2 provides a detailed summary of all outcome measures, as specified in the SPIRIT 2025 statement, including domain, measure, metric, method of aggregation, and timing.

**Table 2:** Detailed specification of outcome measures

Outcome	Domain	Measure	Specific Metric	Method of Aggregation	Timing
Primary	Delirium incidence	DSM-5 criteria	Binary (Yes/No)	Proportion (%)	Within 72 h
Secondary 1	Time-to- delirium onset	DSM-5 criteria	hours	The Kaplan–Meier method	Within 72 h
Secondary 2	Cumulative duration of delirium	DSM-5 criteria	hours	Mean (SD)	Within 72 h
Secondary 3	Peak Delirium severity	DRS-R98-J	Total/severity scores	Mean (SD)	Within 72 h
Secondary 4	Peak sleep–wake cycle	Item 1 of DRS-R98-J	Item score (0–3)	Mean (SD)	Within 72 h
Secondary 5	Rescue medication	Hydroxyzine (mg)	Cumulative dose	Mean (SD)	Within 72 h
Secondary 6	Agitation (Dementia)	Clinical assessment	Presence/absence	Proportion (%)	Within 72 h

### Primary outcome {16a}

The primary outcome will be the incidence of delirium within 3 days of the study initiation. Diagnosis will be established by an independent psychiatrist blinded to group allocation using the Diagnostic and Statistical Manual of Mental Disorders, 5th Edition (DSM-5) criteria. To ensure diagnostic accuracy, the psychiatrist will integrate the direct examination findings, nursing observations (including the DRS-R98-J scores), and baseline mental status reports from families.

### Secondary outcomes {16a}

The secondary outcomes will include the following:

- Time to delirium onset: Measured as the time in hours from study enrollment to the first diagnosis of delirium within 72 h and analyzed using the Kaplan–Meier method.
- Cumulative duration of delirium: Measured as the total number of hours during which the participant met the DSM-5 criteria for delirium, summarized as the mean total duration per participant within the 72-h study period.
- Peak Delirium severity: Assessed using the maximum total and severity scores of the DRS-R98-J, expressed as the maximum score (worst value) recorded over 72 h.
- Peak Sleep–wake cycle disturbances: Assessed using item 1 of the DRS-R98-J, expressed as the maximum score (worst value) recorded over 72 h.
- Rescue medication use: Measured as the total cumulative dose of hydroxyzine, expressed as the mean total dose per participant over 72 h.
- Incidence of restlessness and agitation: The proportion of participants experiencing clinical events associated with dementia within 72 h.

Additionally, a subgroup analysis will be conducted to compare the incidence of delirium based on the specific type of study drug administered. To ensure high inter-rater reliability for the nursing assessments (DRS-R98-J), all participating nurses underwent intensive training using original video-based educational materials prior to the study.

### Harms {17}

#### Methods for collecting harms

Adverse events (AEs) will be collected systematically through daily clinical assessments and medical record reviews by the investigators from days 1 to 4. In addition to pre-specified harms, including hyperglycemia, extrapyramidal symptoms, falls, and oversedation, all unanticipated or unexpected harms will be identified through both daily bedside observations and spontaneous reporting by the nursing staff.

### Classification and grading

To ensure standardized reporting, all AEs will be classified using the Common Terminology Criteria for Adverse Events (CTCAE) version 5.0. The severity of each event will be graded on a scale from 1 (mild) to 5 (death). Safety will be also monitored through daily clinical laboratory tests (days 1–4), including complete blood count, blood chemistry (liver and renal function), and electrolytes, with results compared to baseline values obtained within 7 days before enrollment.

### Reporting and publication

All serious AEs occurring during the 7-day post-intervention period, as well as any event occurring after day 8 that is suspected to be causally related, will be reported to the Institutional Review Board (IRB) and the clinical trial insurance provider. In the final publication, all collected AEs, regardless of their suspected causal relationship to the intervention, will be presented in a summarized table to ensure full transparency of the safety profile of the PBPM.

### Participant timeline {18}

**Fig. 3:**
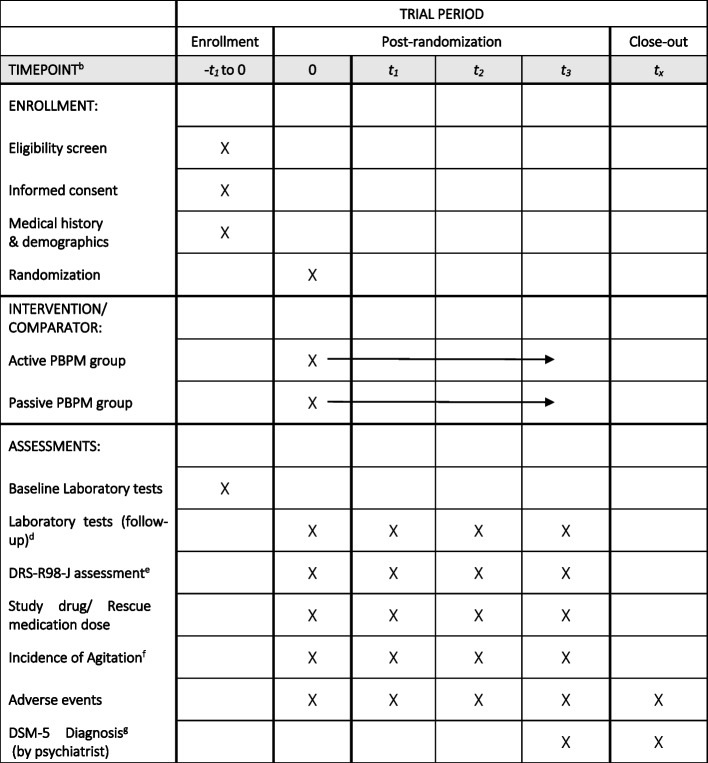
Schedule of enrollment, interventions, and assessments. (a) Recommended content can be displayed using various schematic formats. (b) Time points: −t1 to 0: Enrollment phase; 0: day 1 (initiation of PBPM); t1: day 2; t2: day 3; t3: day 4 morning (end of intervention); tx: day 7 (final follow-up). (c) Arrows indicate continuous delivery of PBPM or standard care. (d) Laboratory tests are performed at least once between day 1 and Day 4. (e) DRS-R98-J: Conducted by trained nurses twice daily (10:00 and 20:00), except day 1 (evening only) and day 4 (morning only). Time window is ±30 min. (f) Agitation is monitored continuously through nursing logs. (g) DSM-5 Diagnosis: Established by a blinded psychiatrist between day 4 and Day 7

The schedule of enrollment, interventions, and assessments is summarized in Fig. 3.

### Sample size {19}

The sample size was determined on the basis of previous studies that investigated delirium prevention using orexin receptor antagonists and melatonin receptor agonists [8, 18]. One study reported a delirium incidence rate of 20.5% in the standard administration group (equivalent to the Passive PBPM group) compared with 7.4% in the early administration group (equivalent to the Active PBPM group). Another randomized controlled trial reported delirium rates of 16.7% and 0% in the placebo and suvorexant groups, respectively.

In this study, we anticipate a higher baseline incidence because the criteria include patients with nil per os status. Therefore, we estimated the incidence rates of delirium within 3 days of admission to be 25% in the Passive PBPM group and 5% in the Active PBPM group. To detect this difference with 80% power at a two-sided significance level of 5% (*α* = 0.05) using Newcombe’s method for the difference in proportions, 96 patients will be required. Accounting for a projected dropout rate of 10%, the target sample size will be set at 105 patients.

### Recruitment {20}

To ensure the target sample size is achieved within the study period, several proactive strategies have been implemented:

1. Expansion of Eligibility and Timing: To maximize the recruitment rate, the protocol was amended to expand enrollment days to include weekends and public holidays. Additionally, the enrollment window was extended from the day of admission (day 1) to the following day (day 2) to capture patients admitted at night or outside regular working hours. (details are provided in Section {31}).
2. Multi-professional Collaboration: The study is conducted through a collaborative effort involving emergency physicians, psychiatrists, pharmacists, and nurses. This team-based approach ensures a 24/7 screening capability and prevents the missing of potential candidates.
3. Pharmacist-led Screening: Pharmacists screen all new admissions to the emergency ward daily, utilizing the hospital’s electronic medical record system to identify eligible patients aged 65 years or older.
4. Ongoing Staff Education: Periodic briefing sessions are provided to the nursing and medical staff to maintain awareness of the study and ensure adherence to the revised enrollment procedures.

## Assignment of interventions: randomization

### Sequence generation: who will generate the sequence {21a}

The randomization sequence will be generated by an independent data center (Department of Clinical Trial Science, iACT, Kyoto University Hospital) using the minimization method. This study will use a Prospective Randomized Open-label Blinded Endpoint design.

### Sequence generation: type of randomization {21b}

**Fig. 4:**
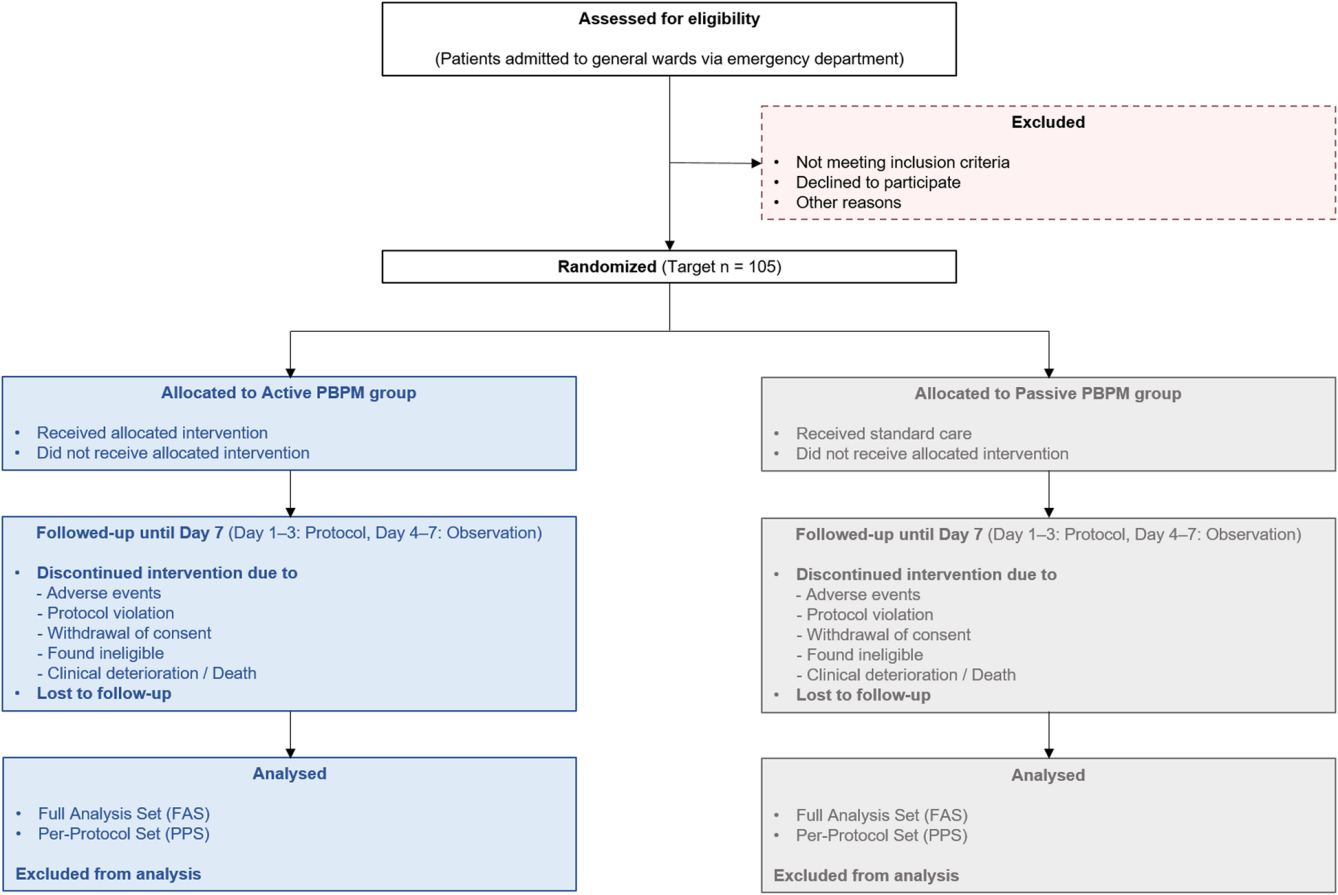
Study flowchart. The participants will be randomized into the Active or Passive PBPM group. In the Active PBPM group, prophylactic medications will be administered based on the ability to take oral intake. In the Passive PBPM group, medications will be administered only when insomnia occurs

Participants will be randomly assigned in a 1:1 ratio to either the Active or Passive PBPM group (Fig. 4). Stratification factors will include age (<75 vs. ≥75 years) and the baseline use of high-risk drugs for delirium.

### Allocation concealment mechanism {22}

Selection bias will be minimized using a sequentially numbered allocation list. After obtaining written informed consent and confirming eligibility, the investigators will reveal the allocation by opening the opaque, tamper-evident seals.

### Implementation {23}

The enrollment and assignment procedures will be conducted as follows to ensure strict adherence to the protocol:

1. Enrollment: Emergency physicians will screen eligible patients and obtain written informed consent.
2. Assignment: After confirming eligibility, the ward physician (or the ward physician in collaboration with the ward pharmacist) will open the next sequentially numbered, opaque, tamper-evident seal to reveal the group assignment.
3. Communication and Initiation: The assignment results will be shared among the ward physicians, nurses, and pharmacists. Based on the revealed group (Active or Passive PBPM), the ward physician and pharmacist will initiate the intervention by providing medical orders to the nurses. The ward nurses will then administer the medications or apply the patches to the patient according to these orders. The personnel responsible for the initial enrollment (emergency physicians) will not have access to the allocation sequence in advance, and the assignment will remain concealed until the point of opening the seal.

### Assignment of interventions: blinding

#### Who will be blinded {24a}

Owing to the nature of the pharmacological intervention, the patients and ward staff will not be blinded. However, the psychiatrists responsible for the definitive diagnosis of delirium (the primary outcome) will be strictly blinded to the group allocation to prevent detection bias.

### How will be blinding be achieved {24b}

As this study evaluates a pharmacological management protocol involving different administration timings (prophylactic vs. symptomatic), it is conducted as an open-label trial, and no placebo will be used. Therefore, the ward physicians, pharmacists, and nurses will not be blinded to the group assignment. However, to minimize detection bias, the primary outcome (diagnosis of delirium based on DSM-5 criteria) will be assessed by psychiatrists who are not involved in the participants’ daily care or the PBPM intervention. The group assignment is not formally disclosed to these psychiatrists, and they establish the diagnosis as independent evaluators based on the clinical records. This approach follows the PROBE (Prospective Randomized Open-label Blinded Endpoint) design.

### Procedure for unblinding if needed {24c}

Not applicable. This is an open-label trial in which ward physicians, pharmacists, and nurses are aware of the group assignment from the time of enrollment. Therefore, no specific procedure for unblinding is required in the event of an emergency, as the intervention details are already known to the clinical staff responsible for the participant’s safety.

## Data collection and management

### Plans for assessment and collection of outcomes {25a}

Data will be collected and managed to ensure high quality and reliability through the following plans:

1. Clinical Assessment Tools: The primary and secondary outcomes will be assessed using the DRS-R98-J, a validated Japanese version of a widely used tool for evaluating delirium severity and diagnosis with high inter-rater reliability.
2. Training of Assessors: Before study initiation, all participating nurses will receive standardized training on the use of the DRS-R98-J to minimize inter-observer variability. The psychiatrists responsible for the final diagnosis are experts in delirium management and follow the DSM-5 criteria.
3. Data Collection and Quality Assurance: Data will be initially recorded in the electronic medical records and then transcribed into paper-based case report forms (CRFs) by the investigators. The principal investigator will ensure the completeness and accuracy of all entries. To maintain data integrity, a double-check against source documents will be performed, and any corrections to the CRFs will be documented according to standardized procedures.

### Plans to promote participant retention and complete follow-up {25b}

Given the acute nature of the setting and the short follow-up period (72 h), high participant retention is expected. To ensure complete follow-up and minimize bias, the following strategies will be employed:

1. Retention Strategies: The PBPM intervention and assessments will be integrated into the standard clinical workflow of the emergency ward to minimize the burden on participants. Investigators and ward staff will monitor the participants’ clinical status daily to ensure adherence to the protocol.
2. Data Collection for Discontinued Participants: If a participant deviates from or discontinues the intervention protocol (e.g., due to medical necessity, adverse events, or transfer to another ward), every effort will be made to continue the outcome assessments until the 72-h endpoint, provided that consent is not withdrawn.
3. Outcome Data for Deviations: For participants who discontinue the study, the following data will be collected up to the point of discontinuation and included in the intention-to-treat (ITT) analysis:
  DRS-R98-J
  Incidence of agitation and medication records
  Final delirium diagnosis by a psychiatrist (if possible, based on records up to the time of discontinuation)
4. Withdrawal of Consent: If consent is withdrawn by the participant or their representative, all further data collection will cease. Data collected up to that point will be handled in accordance with the ethical approval and the participant’s preference.

### Data management {26}

Data management will be performed by Takahiko Tsutsumi (TT). On-site monitoring will be conducted annually by Yoshinori Okuno (YO) to ensure data integrity and protocol compliance. Although both are affiliated with the Department of Primary Care and Emergency Medicine at Kyoto University Hospital, they are strictly independent of the study execution team and will not be involved in patient enrollment, allocation, or clinical assessment. This oversight structure ensures objective quality control and rigorous adherence to the study protocol.

### Confidentiality {33}

Access to the raw data will be restricted to authorized study personnel only. All data will be maintained in accordance with the CRA and the study protocol.

## Statistical methods

### Statistical methods for primary and secondary outcomes {27a}

The primary analysis will be performed on the FAS and repeated on the PPS as a sensitivity analysis. The incidence of delirium diagnosed using the DSM-5 within 3 days will be calculated for each group, with 95% confidence intervals (CIs). The between-group difference in incidence (Active PBPM minus Passive PBPM) and its 95% CI will be estimated using Newcombe’s method. Statistical significance will be set at a two-sided level of 5%. If the upper limit of the 95% CI for the difference is <0, the Active PBPM group will be considered to have a significantly lower incidence of delirium.

Unless otherwise specified, following secondary analyses will use a two-sided significance level of 5% and 95% CIs.

- Time to delirium: The time from enrollment to the onset of delirium (DSM-5) will be analyzed using Kaplan–Meier curves, with observations censored at 72 h.
- Delirium severity and sleep–wake cycle: Summary statistics for the worst DRS-R98-J severity scores and item 1 (sleep–wake cycle disturbance) scores recorded while the patient was awake will be compared between groups.

### Who will be included in each analysis {27b}

The analysis populations will be defined as follows:

- Safety analysis set: All registered participants who received at least one dose of the study drug.
- Full analysis set (FAS): All registered participants who received at least one dose of the study drug, excluding those with major protocol violations (e.g., eligibility criteria violations, failure to obtain consent, non-registration, or other significant procedural errors).
- Per-protocol set (PPS): Participants in the FAS who strictly adhered to the 3-day intervention protocol and had complete data on the primary outcome.

### How missing data will be handled in the analysis {27c}

Missing data are expected to be minimal given the short follow-up period of 72 h. The primary analysis will be based on available cases in the FAS. However, if the proportion of missing data for the primary outcome exceeds 10%, a sensitivity analysis using worst-case scenario imputation will be performed to assess the robustness of the findings. Regarding time-to-delirium onset, participants who are discharged or die before 72 h without delirium will be treated as censored.

### Methods for additional analyses (e.g., subgroup analyses) {27d}

Between-group differences in the incidence of agitation, behavioral and psychological symptoms of dementia (BPSD), and delirium incidence according to specific study drugs will be assessed using Newcombe’s method. The total dose of the rescue medication (hydroxyzine) will be summarized for each group.

### Interim analyses {28b}

No interim analyses are planned for this study. The trial will not be terminated early for efficacy. However, for safety monitoring, the principal investigator will continuously review all adverse events and serious adverse events, formally documenting any safety concerns identified during this process. The principal investigator, in consultation with the independent data center, may decide to terminate the trial prematurely if any unexpected and significant safety concerns arise or if the risk–benefit balance is deemed unfavorable. All such decisions, along with the underlying safety data will be reported to the IRB. The trial may also be terminated if recruitment becomes unfeasible.

### Protocol and statistical analysis plan {5}

This document serves as the primary protocol for the study. The detailed statistical analysis plan (SAP) is integrated within this protocol (see Statistical Methods {27}). The trial is registered at the Japan Registry of Clinical Trials (jRCT), and the registry information is available at jRCTs051220122. Any subsequent major updates or amendments to the protocol and SAP will be submitted as an update to this journal and updated in the jRCT.

## Oversight and monitoring

### Composition of the coordinating centre and trial steering committee {3d}

As this is a single-center study, the trial is managed with a streamlined structure to ensure quality and oversight:

1. Trial Management Group (TMG): Composed of the principal investigator and co-investigators. They are responsible for the daily execution of the trial, including recruitment and protocol adherence. They meet annually (or more frequently as needed) to review the study’s progress.
2. Data Coordinating Center: This center is responsible for generating the randomization sequence and allocation list. To ensure data integrity, they will provide monthly monitoring of the registration status and data entry throughout the trial period.
3. Institutional Support: The Institute for Advancement of Clinical and Translational Science (iACT) provided expert consultation and administrative support during the study development phase, including advice on protocol design and the ethical review process.

### Composition of the data monitoring committee, its role and reporting structure {28a}

No formal data monitoring committee (DMC) was established for this study. As this is a single-center investigator-initiated trial with a short intervention period (72 h) and the pharmacological interventions are within the scope of standard clinical practice, the risks to participants are considered minimal. Instead, safety and trial progress will be closely monitored by the principal investigator and the Trial Management Group. Additionally, the Data Coordinating Center will perform monthly monitoring of registration and data entry to ensure study integrity.

### Frequency and plans for auditing trial conduct {29}

To ensure the highest standards of study conduct and data integrity, the trial will be subject to regular monitoring and auditing as follows:

1. External Monitoring: An independent external monitor (not involved in the daily conduct of the trial) will perform an audit/monitoring visit once a year. This process includes verifying informed consent documentation, assessing adherence to the inclusion/exclusion criteria, and cross-checking the CRFs against source medical records (Source Data Verification).
2. Internal Review: The Trial Management Group will conduct ongoing internal reviews of study procedures. Additionally, the Data Coordinating Center will provide monthly reports on the status of participant registration and data entry, allowing for the early detection of any procedural deviations.
3. Audit Independence: The audit process will be independent of the investigators and the study funder to ensure objective oversight.

### Protocol amendments {31}

To ensure recruitment feasibility within the designated study period, the inclusion criteria and enrollment procedures were amended twice following study initiation. All changes were approved by the Institutional Review Board of Kyoto University Hospital.

1. First Amendment (June 21, 2024, Version 4.0): Expansion of recruitment days. Initially, enrollment was restricted to patients admitted on weekdays (8:30–17:00). To improve the recruitment rate, this restriction was removed, allowing enrollment on weekends and public holidays within the same time window.
2. Second Amendment (April 9, 2025; Version 5.0): Extension of the enrollment window. To accommodate patients admitted outside of regular working hours or at night, the enrollment window was expanded from the day of admission (day 1, until 17:00) to the following day (day 2, until 17:00). To maintain the integrity of the intervention, patients enrolled on day 2 will be excluded if they have already received any study medications (ramelteon, lemborexant, or blonanserin patch), rescue medications (hydroxyzine), or antipsychotics before enrollment.

The target sample size remained unchanged following these amendments.

## Dissemination policy {8}

The results of this study will be disseminated to ensure transparency and contribute to the improvement of clinical practice:

- Peer-reviewed Publications and Presentations: The findings will be submitted for publication in peer-reviewed international journals. Additionally, results will be presented at relevant domestic and international conferences (e.g., emergency medicine or psychiatry societies).
- Trial Registry: Within 1 year of study completion, a summary of the results will be reported on the Japan Registry of Clinical Trials (jRCT) website, which is accessible to the public.
- Communication with Participants: Since it may be difficult to contact participants directly after discharge, the results will be made available through public reporting and publications. If requested by a participant or their representative, an explanation of the study findings will be provided.
- Authorship: Authorship for publications will be determined based on the International Committee of Medical Journal Editors (ICMJE) recommendations.

## Discussion

This study addresses a critical gap in the management of older patients in emergency settings. Although the efficacy of individual pharmacological agents has been studied, the effectiveness of a system-based approach, such as PBPM, for prophylaxis is not well established. If the Active PBPM strategy proves effective, it can provide a practical model for delirium prevention that can be implemented in acute care hospitals, leveraging the expertise of pharmacists to reduce the burden on physicians and improve patient outcomes. One limitation of this study is its single-center, open-label design. However, diagnosis by psychiatrists (blinded to allocation) using the DSM-5 criteria and the use of a validated scale (DRS-R98-J) by nurses trained with standardized video materials help mitigate bias.

## Trial status

The current protocol version is 6.0, dated May 26, 2025. Recruitment began on November 25, 2022. Recruitment is currently ongoing and is expected to be completed by February 28, 2026. The final study completion is scheduled for March 31, 2026.

## Data Availability

The datasets used in this study are available from the corresponding author upon request.

## Abbreviations

AE: Adverse event
BPSD: Behavioral and psychological symptoms of dementia
CI: Confidence interval
CRA: Clinical Research Act
DSM-5: Diagnostic and Statistical Manual of Mental Disorders, 5th Edition
DRS-R98-J: Delirium Rating Scale-Revised-98, Japanese version
eGFR: Estimated glomerular filtration rate
FAS: Full analysis set
HCU: High-care unit
iACT: Institute for Advancement of Clinical and Translational Science
ICU: Intensive care unit
PBPM: Protocol-Based Pharmacotherapy Management
PPS: Per-protocol set
